# Effects of Cu Addition on Age Hardening Behavior and Mechanical Properties of High-Strength Al-1.2Mg-1.2Si Alloy

**DOI:** 10.3390/ma16083126

**Published:** 2023-04-15

**Authors:** Xu Zhang, Lizhen Yan, Zhihui Li, Xiwu Li, Guanjun Gao, Hongwei Yan, Kai Wen, Yongan Zhang, Baiqing Xiong

**Affiliations:** 1 State Key Laboratory of Nonferrous Metals and Processes, China GRINM Group Co., Ltd., Beijing 100088, China; 2 GRIMAT Engineering Institute Co., Ltd., Beijing 101407, China; 3 General Research Institute for Nonferrous Metals, Beijing 100088, China

**Keywords:** Al-Mg-Si-(xCu) alloy, age hardening behavior, mechanical properties, TEM observation and analysis, strengthening precipitates

## Abstract

In this study, the effects of Cu addition on artificial age hardening behavior and mechanical properties of Al-1.2Mg-1.2Si-(xCu) alloy was investigated quantitatively and qualitatively by Vickers hardness, tensile test, and transmission electron microscope. The results indicated that Cu addition enhanced the aging response of the alloy at 175 °C. With the increase in Cu content, the time for the alloys to reach peak aging decreased from 12 h to 10 h and 8 h. The tensile strength of the alloy was obviously improved with Cu added in which was 421 MPa of 0Cu alloy, 448 MPa of 0.18Cu alloy, and 459 MPa of 0.37Cu alloy. The results of TEM observation revealed that the addition of 0.37Cu changed the aging precipitation sequence of the alloy, in which the precipitation sequence of 0Cu and 0.18Cu alloy was SSSS→GP zones/pre-β″→β″→β″ + β′, 0.37Cu alloy was SSSS→GP zones/pre-β″→β″ + L→β″ + L + Q′. Moreover, with the addition of Cu, the number density and volume fraction of precipitates of the Al-1.2Mg-1.2Si-(xCu) alloy was evidently increased. The number density was increased from 0.23 × 10^23^/m^3^ to 0.73 × 10^23^/m^3^ in the initial aging stage and from 1.9 × 10^23^/m^3^ to 5.5 × 10^23^/m^3^ in the peak aging stage. The volume fraction was increased from 0.27% to 0.59% in the early aging stage and from 4.05% to 5.36% in the peak aging stage. It indicated that Cu addition promoted the precipitation of strengthening precipitates and boosted the mechanical properties of the alloy accordingly.

## 1. Introduction

In the field of transportation, with the increase in lightweight demand, lightweight materials have been widely concerned by people. Although magnesium alloy is the lightest structural alloy, it has the disadvantages of poor corrosion resistance and insufficient ductility [1,2,3]. Aluminum alloys such as Al-Mg-Si have gradually become one of the highest quality lightweight materials due to their excellent comprehensive properties of light weight, medium strength, good welding performance, and corrosion resistance. It was widely used in the automotive and aerospace industry [4]. Compared with Al-Zn-Mg alloy, Al-Mg-Si has higher corrosion resistance, but lower strength. Automobile extrusions such as thrust rods and bumpers, not only have requirements for corrosion resistance but also has certain requirements for strength. The commonly used extrusion Al-Mg-Si alloys such as 6005 are not strong enough to be applied to these structures, which require a higher strength for Al-Mg-Si alloys to meet the development needs.

In order to improve the properties of the alloy, the researchers began microalloying the Al-Mg-Si alloy with the addition of microalloying elements such as Cu, Ag, Zr, Sc, and so on. In the past, many researchers found that adding Cu or Ag to Al-Mg-Si alloys can significantly improve the Vickers hardness and strength of the alloy and they can promote the nucleation of β″ phase, making the precipitates in the aging process more dispersed and finer [5,6,7,8,9]. Furthermore, the addition of Cu may also cause the Q phase to be formed during the aging process [10], which is a key strengthening phase in aluminum alloys. The addition of Zr or Sc can significantly refine the as-cast structure, and the refining effect was stronger when they were added together [11,12,13]. Moreover, Zr participated in the nucleation of β″ and promoted the precipitation of the enhanced phase. Consequently, Zr can improve the strength of the aging alloy. In addition to the reinforcement, the addition of Sc can optimize the mechanical properties of the aluminum alloy at high temperatures [14,15]. Although the addition of Ag, Zr, and Sc can improve the strength, Ag-added, Zr-added or Sc-added alloys are not suitable for industrial mass production because of their valuableness. Therefore, under the comprehensive consideration of various factors, adding Cu is undoubtedly one of the best means to improve the properties of the alloy.

The strengthening mechanisms of conventional alloys mainly included grain boundary strengthening, solid solution strengthening, dislocation hardening, and precipitation hardening [16]. As an age-hardenable alloy, the strength of Al-Mg-Si alloy was based on the strengthening effect of the formation of precipitates during artificial aging [17]. Consequently, studying the precipitates during artificial aging was crucial to understanding the strengthening mechanism of alloys. Generally, the precipitation sequence of Al-Mg-Si alloy was: supersaturated solid solution (SSSS)→Guinier-Preston (GP) zones→β″→β′→β [18,19,20,21]. The needle-like β″ phase was the most important precipitated strengthening phase in the Al-Mg-Si alloy [22,23]. It was mainly formed after the peak aging of the alloy. When Cu was added to Al-Mg-Si alloy, the precipitation sequence became: SSSS→GP zones→β″, L, C, QP, QC→β′, Q′→Q [24,25,26,27,28,29,30,31,32,33]. In those studies, the crystal parameters and orientation relationship (ORs) of precipitates were studied, and the detailed information is displayed in Table 1.

Although there were many studies on the addition of Cu to Al-Mg-Si alloy, the effect of Cu addition on the precipitation sequence of Al-Mg-Si alloy was very complicated. In this study, a new Al-1.2Mg-1.2Si-(xCu) alloy with tensile strength higher than 420 MPa was selected as the base alloy. The artificial aging hardening behavior and mechanical property of the alloys with different Cu contents were studied quantitatively and qualitatively. The number density and volume fraction of precipitates at different aging stages were quantitatively analyzed. Through this research, we established the relationship between microstructure and mechanical properties of Al-1.2Mg-1.2Si-(xCu) alloys. Furthermore, the effect of Cu added on the mechanism of aging strengthening to Al-1.2Mg-1.2Si-(xCu) alloy was analyzed from the atomic scale.

## 2. Materials and Experimental Methods

### 2.1. Materials and Thermal Treatments

The investigations were carried out on hot-extruded Al-1.2Mg-1.2Si-(xCu) alloy plates with the chemical composition as shown in Table 2. The chemical composition measurement was taken by inductively coupled plasma atomic emission spectrometry (ICP-AES). The extruded plate had a shape of 62 mm in L (longitudinal direction) and 16 mm in T (long transverse direction). The main difference between the alloys was the difference in Cu element. Specimens of microstructure observation and property tests were cut from the center of cross-section and paralleled to the extrusion direction. The specimens were solution heat treated with a regime of 555 °C/2 h and 540 °C/2 h in a KU70 muffle furnace and water quenched. Then, the alloys were preserved at a typical temperature of 175 °C from 0 h to 72 h in a thermostatic drier box.

### 2.2. Experimental and Performance Test Methods

The age hardening process was monitored by a 430SVD Vickers hardness tester using a loading force of 5 kgf and a dwelling time of 15 s. Each Vickers hardness datum of the sample was the mean value of five indentations, which was calculated after rounding a maximum and a minimum from seven test data. The size of the Vickers hardness sheet was 20 mm × 20 mm × 3 mm (L × T × S). The tensile tests were carried out on the MTS-WD3100 testing machine, and the experiments were carried out in accordance with the standard of ISO 6892. The samples with a size of M10-Φ5 for tensile test were cut from the plates paralleling the extrusion direction. In the tensile tests, we carried out three tests for each alloy. Three parallel samples were included in each alloy. Considering that there was little difference in the property of each parallel sample, the data of one sample on each alloy was selected to draw the stress–strain curve.

### 2.3. Microstructural Characterization and Statistics

The main characterization method in this study was transmission electron microscope (TEM) and high-resolution transmission electron microscopy (HRTEM). It was performed using Tecnai G2 F20 that operated at 200 kV. The results of the observation will be analyzed by Image Pro Plus and Gatan Digital Micrograph software.

#### 2.3.1. Preparation of TEM Film

TEM film specimens with a thickness of 50~60 μm were prepared on the twin-jet electropolishing device. Their diameters were 3 mm. The electrolyte was consisting of 30 vol% nitric acid and 70% methanol. The operating voltage of this experiment was about 15 V and the operating temperature was about −30 °C. After the experiment, the samples should be quickly put into methanol and ethanol solution for cleaning. Then, the liquid on the surface of the sample should be blotted with filter paper and placed in the sample box for subsequent observation experiments.

#### 2.3.2. TEM Film Thickness Test Method

TEM film thickness was mainly measured by converged beam electron diffraction (CBED) method. This test method calculated the offset vector *Δθi*: for each fringe by calibrating the difference in the Bragg angle of the light and dark fringes in the CBED diffraction disk under the condition of dual beam [34]:

(1) $$S_{i}=\lambda \frac{\Delta \theta_{i}}{2\theta_{B}d^{2}}$$

where *S_i_* was the deviation vector value of diffraction, *θ_B_* was the Bragg diffraction angle of the HKL crystal face, d was the interplanar crystal spacing of the HKL crystal face, *λ* was the electron wavelength, and *n_i_* was the integer. After that, it depended on the relationship between *S_i_*, extinction distance *ξ_g_*, and sample thickness *t* [34]:

(2) $$\frac{S_{i}^{2}}{n_{i}^{2}}+\frac{1}{\xi_{g}^{2}n_{i}^{2}}=\frac{1}{t^{2}}\;$$

**Figure 1:**
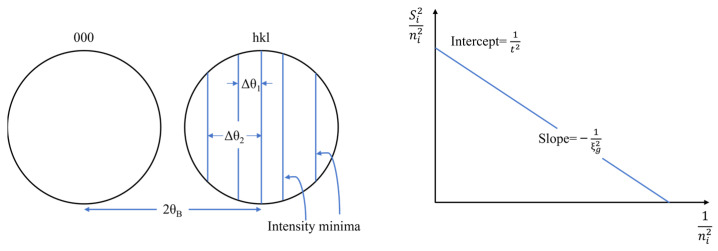
Schematic diagram of film thickness measurement by CBED method [34].

After that, linear fitting was performed with $\frac{S_{i}^{2}}{n_{i}^{2}}$ and $\frac{1}{n_{i}^{2}}$. The method of measuring film thickness and the fitted straight line is shown in Figure 1. It should be noted that in order to reduce the error of measurement, the number of light and dark fringes which can be observed in the diffraction disk should be ensured three pairs at least.

#### 2.3.3. Statistical Methods for Precipitated Phases

The statistical information of the spherical GP zones was the number density and volume fraction of precipitates. For the statistics of average diameter of the GP zones, the corresponding statistical area needed to be colored in advance through the Photoshop software, and then the colored photo should import into Image Pro Plus. The corresponding statistics were carried out in the software to obtain the average diameter $\bar{d}$ of the GP zones, so as to obtain the volume of GP zones [35]:

(3) $$\bar{V}=\frac{\pi }{6}{\left(\bar{d}\right)}^{3}$$

then the number density of GP zones was [35]:

(4) $$\rho =\frac{N/V}{1+d/t}$$

where A was the area of the TEM image and V was the product of A and film thickness t (V = At). N was the number of GP zones observed in TEM image. Then the volume fraction of the GP region was [35]:

(5) $$f=\rho \bar{V}$$

β″ phase was an important precipitation phase in 6xxx series aluminum alloys. The aging precipitation was a three-dimensional process. Therefore, in the characteristic of TEM, it was necessary to count the quantity (N), and size (l) of precipitates. If only calculated the two-dimensional information, there was a certain error. Hence it was also necessary to perform statistical and quantitative analysis of the three-dimensional information of the precipitates, such as quantity density (n) and volume fraction (V). The statistical analysis of the information needed to be combined with TEM brightfield image and TEM film thickness and obtained through certain calculations.

In the above information, the statistics of the size were mainly based on the average length and average cross-sectional of the needle-like β″ phase in the 〈100〉_Al_ in the brightfield image. 

The number density of β″ phases was calculated by total amount N of precipitates, image area A, and film thickness t in the bright field image. The calculation formula was as follows [35]:

(6) $$n=\frac{3N}{At\left(1+\frac{l}{t}\right)}$$

The volume fraction of the β″ phase was calculated as follows [35]:

(7) V=Cln

where *C* was the average cross-sectional area of β″ along the <100>_Al_ direction, which can be measured directly from the TEM image. In the above statistics section, it was essential to pay attention to the correct selection of the ruler of the picture. In addition, in order to reduce the error, the number of precipitates should be ensured over 100 in the selected brightfield image.

## 3. Results

### 3.1. Mechanical Properties

#### 3.1.1. Artificial Age-Hardening Curve

**Figure 2:**
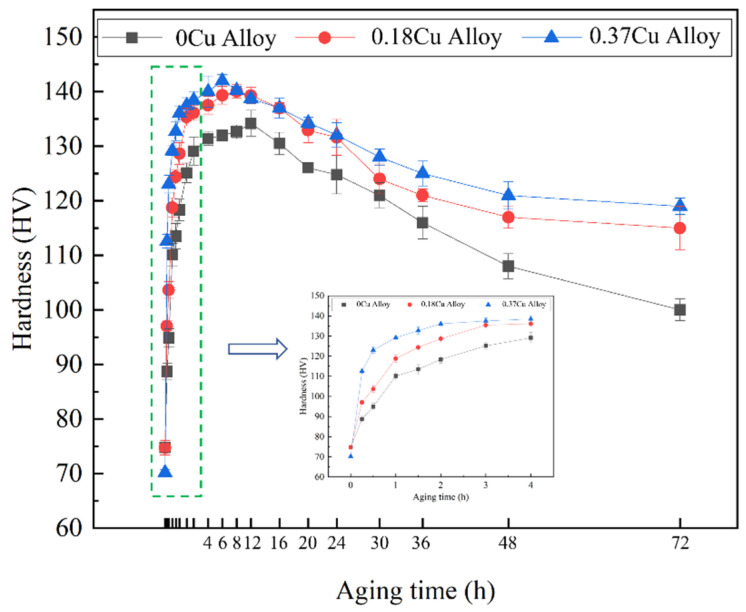
Artificial aging hardening curve at 175 °C.

Figure 2 exhibited the aging hardening curve of three alloys measured at a typical aging temperature of 175 °C, and the aging holding time was 0~72 h. From the aging hardening curve, it can be found that the Vickers hardness of the alloys after the corresponding solution treatment and water quenching was about 70 HV. In addition, the Vickers hardness of the alloy increased significantly with the extension of the aging time. After the aging time reached 1 h, the growth rate of Vickers hardness began to slow down. Additionally, the three alloys would reach peak time when the aging time was 12 h, 10 h, and 8 h, respectively. The peak Vickers hardness of the alloys was 134 HV, 140 HV, and 142 HV, respectively. After peak aging, the 0.18Cu alloy went through a relatively gentle stage, which was the plateau period of peak aging. While 0Cu and 0.37Cu alloy entered the over-aging stage directly after the peak aging. After peak aging, the alloys entered the over-aging stage, and their Vickers hardness began to decrease gradually. In the aging process, it can be seen that the aging hardening response speed of the alloy was accelerated, and the Vickers hardness of the alloy was obviously promoted with the increase in Cu. Additionally, the time for the alloy to reach the peak aging was shortened, and the corresponding peak Vickers hardness increased. The Vickers hardness of the alloys at different aging stages is shown in Table 3. Then, the precipitation behavior of the alloys after initial aging (175 °C/0.5 h), peak aging (175 °C/12 h, 175 °C/10 h, 175 °C/8 h) and over-aging (175 °C/72 h) were studied.

#### 3.1.2. Tensile Properties

**Figure 3:**
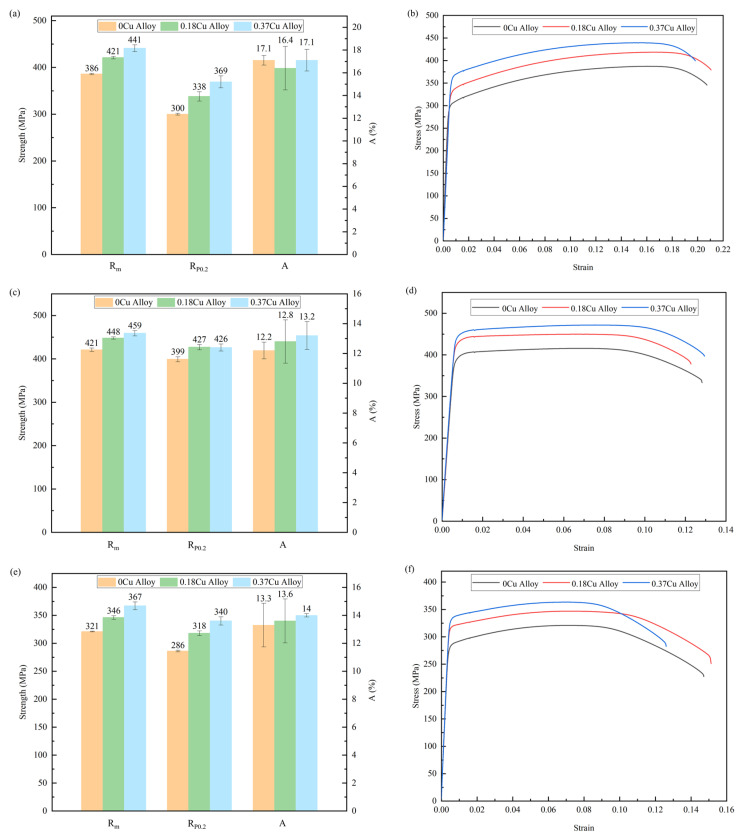
Tensile properties of three alloys at different aging stages. (**a**,**b**) Initial aging; (**c**,**d**) peak timeliness; (**e**,**f**) over-aging.

The tensile properties of the three alloys at different aging stages were tested separately. The test results and stress–strain curves of the alloys are shown in Figure 3. It can be seen that at the same aging stage, with the increase in Cu content, the strength increased significantly. During the aging process, the strength of the alloys increased first and then decreased. At the initial aging stage, the strength of 0.18Cu and 0.37Cu alloy exceeded 420 MPa, while the strength of 0Cu alloy was only 386 MPa. At the peak aging stage, the strength of the alloy increased to more than 420 MPa and decreased to lower than 370 MPa at the over-aging stage. The influence of Cu addition on the tensile strength of the alloy at three aging stages was consistent with that of Vickers hardness. The stress–strain curves of Al-1.2Mg-1.2Si-(xCu) alloys were continuous and smooth. At the initial stage of deformation, the stress of the alloys increased sharply with the increase in strain. Then, the stress increased slowly to the limit of strength, and necking began. There was no obvious yield terrace in the curves. Compared to the stress–strain curves at different aging stages, it can be found that the strength of the alloy increased with the increase in Cu. As shown in Table 4, the mechanical properties between the studied alloys and other extruded Al-Mg-Si alloys which were commonly used were compared. After heat treatment, the tensile strength of the base alloy was about 30 MPa higher than 6013 and 6056 alloys [36].

### 3.2. Microstructural Characterization

#### 3.2.1. Precipitates of Alloys during Initial Aging Stage

**Figure 4:**
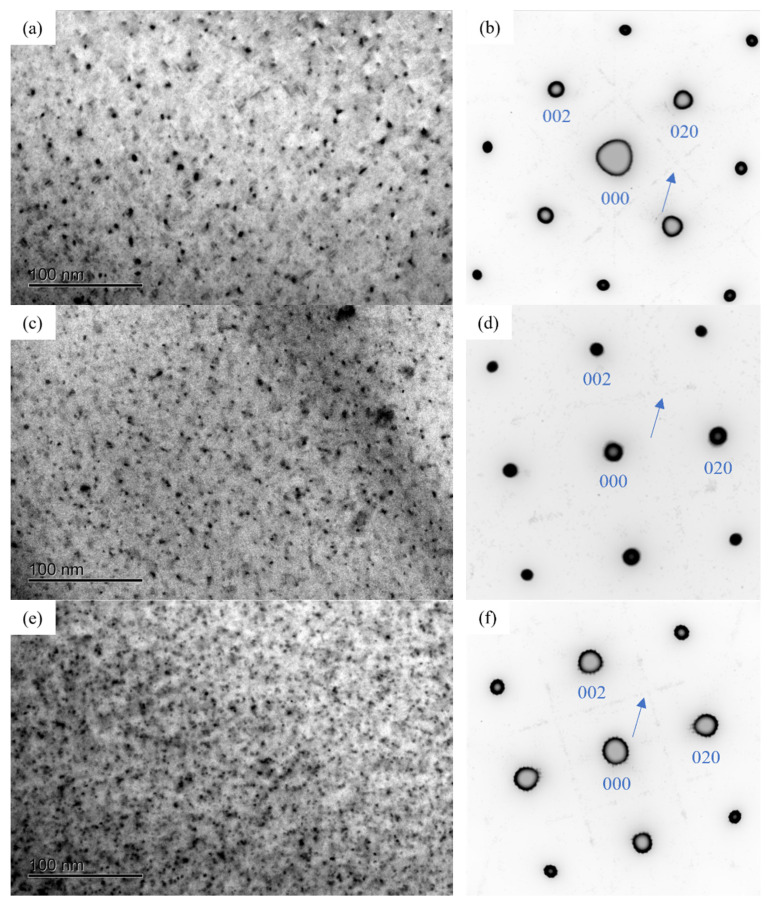
Bright field (BF) image and selected area electron diffraction (SAED) patterns of the alloy after initial aging at 175 °C for 0.5 h of three alloys. (**a**,**b**) 0Cu alloy; (**c**,**d**) 0.18Cu alloy; (**e**,**f**) 0.37Cu alloy.

Figure 4 displayed the TEM bright field (BF) images and the selected area electron diffraction (SAED) patterns of the alloy at the initial aging stage. It can be seen that the morphology of precipitates in the three alloys was mainly spherical shape. In addition, the diffraction streaks aligned along the 〈100〉_Al_ direction were observed in the SAED patterns of the alloys. Compared to the BF images, it can be found that the number of precipitates of 0.37Cu alloy was more than that of 0Cu and 0.18Cu alloy.

**Figure 5:**
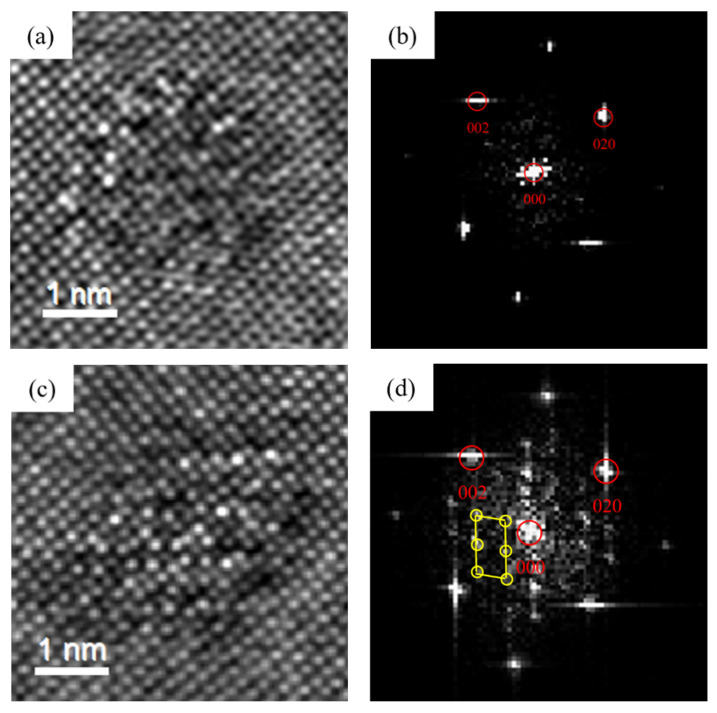
HRTEM and fast Fourier transform (FFT) patterns of precipitates of 0Cu alloy after initial aging at 175 °C for 0.5 h. (**a**,**b**) GP zones; (**c**,**d**) pre-β″.

**Figure 6:**
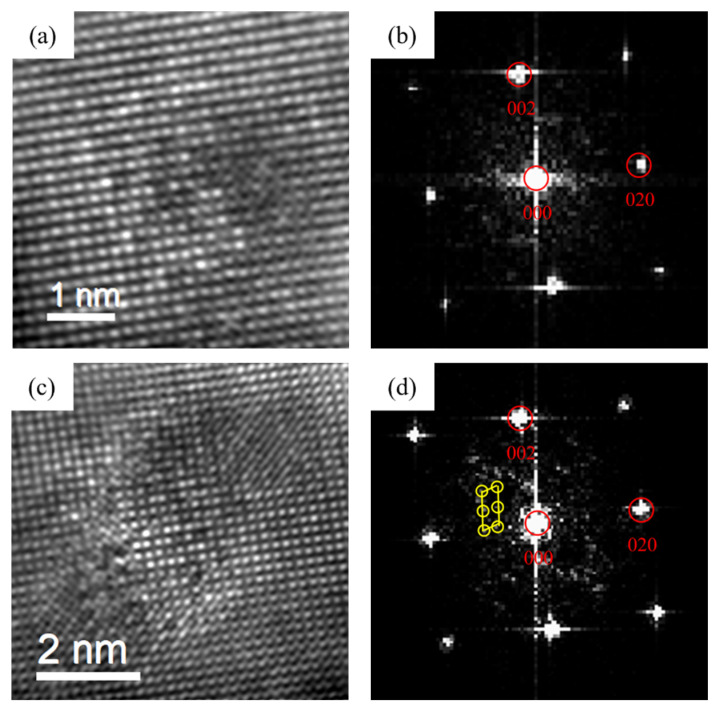
HRTEM and FFT patterns of precipitates of 0.18Cu alloy after initial aging at 175 °C for 0.5 h. (**a**,**b**) GP zones; (**c**,**d**) pre-β″.

**Figure 7:**
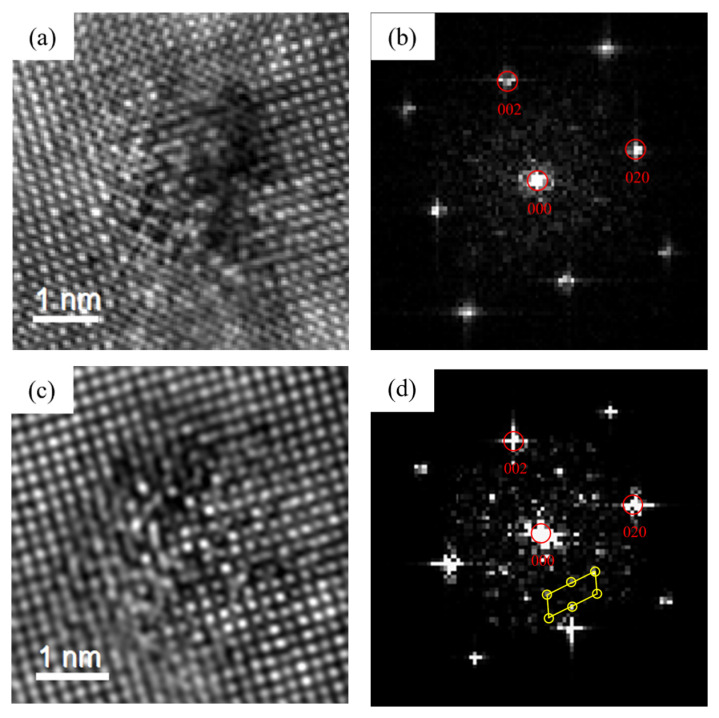
HRTEM and FFT patterns of precipitates of 0.37Cu alloy after initial aging at 175 °C for 0.5 h. (**a**,**b**) GP zones; (**c**,**d**) pre-β″.

The precipitates of the alloys after initial aging were characterized. As shown in Figure 5, Figure 6 and Figure 7, the type of precipitates can be determined according to the HRTEM and fast Fourier transform (FFT) patterns. It can be found that after the artificial aging treatment at 175 °C for 0.5 h, the precipitates in the alloy mainly contained two types. One was coherent with the Al matrix, and there was no extra reflection in the corresponding FFT pattern. It was the same as the study of Thronsen E [37,38], therefore it was determined as GP zones. Others had a crystal parameter with a = 1.516 nm, c = 0.674 nm, and ORs with γ = 105.26°. It was parallel to 〈320〉_Al_ and 〈310〉_Al_. The results were in good agreement with the studies of Andersen S J [22] and Zheng Y Y [23], and it was determined to be the pre-β″ phase.

Table 5 shows the GP zones statistical information of the three alloys after initial aging for 0.5 h at 175 °C. The results indicated that the average diameter of GP zones was 2 to 3 nm. With Cu content increased, so did the number density and volume fraction of GP zones increase. Especially when the Cu content increased from 0.18 to 0.37, the number density and volume fraction of GP zones of the alloy increased significantly. Therefore, when the mass fraction of Cu increased to 0.37, the alloy exhibited the highest Vickers hardness and strength. As shown in Figure 2 and Figure 3a, the mechanical properties of the alloys were significantly improved with the increasing of Cu at the initial aging stage. These indicated that Cu promoted the precipitation of GP zones. GP zones were one of the most important strengthening phases in the early aging process. Therefore, the mechanical property of Al-1.2Mg-1.2Si-(xCu) alloys was enhanced with the increase in the quantity of the GP zone at the initial stage.

#### 3.2.2. Precipitates of Alloys at Peak Aging

**Figure 8:**
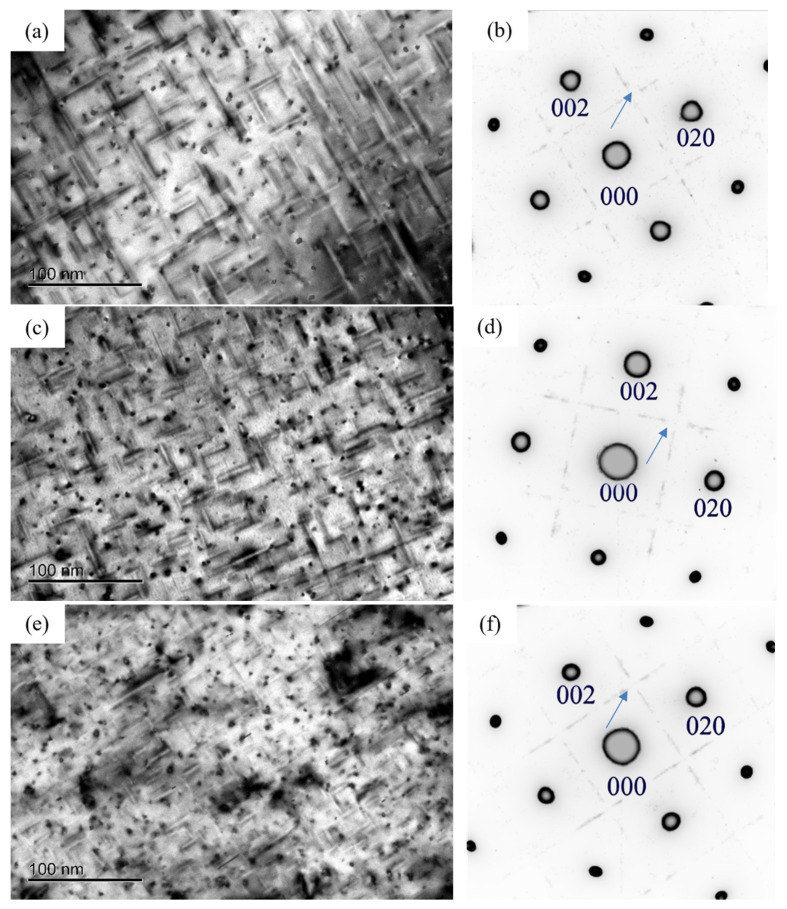
BF image and SAED patterns after peak aging of three alloys. (**a**,**b**) 0Cu alloy; (**c**,**d**) 0.18Cu alloy; (**e**,**f**) 0.37Cu alloy.

Figure 8 shows the BF images and SAED patterns of the three alloys at the peak aging stage. It can be found that a large number of needle-like precipitates grew along the direction of 〈100〉_Al_ in the alloy. In addition to the diffraction spots of the Al matrix, the diffraction streaks aligned along the 〈100〉_Al_ direction were obviously observed in the SAED patterns of the alloys. The BF images showed that the size of precipitates decreased and the number of precipitates increased with the increase in Cu at the peak aging stage. In addition, compared with the initial aging stage, the size of precipitates at peak aging increased significantly.

**Figure 9:**
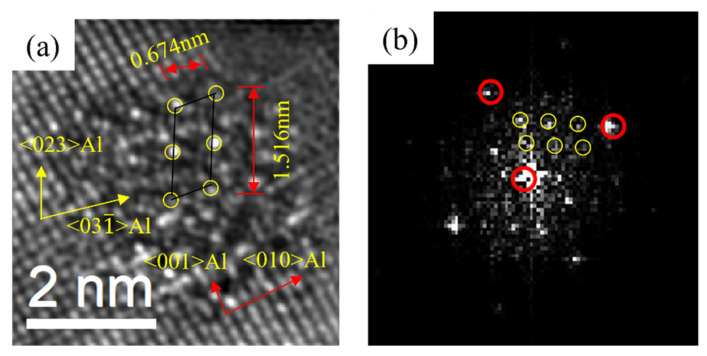
HRTEM (**a**) and FFT (**b**) patterns of the β″ phase of 0Cu alloy after peak aging at 175 °C for 12 h.

**Figure 10:**
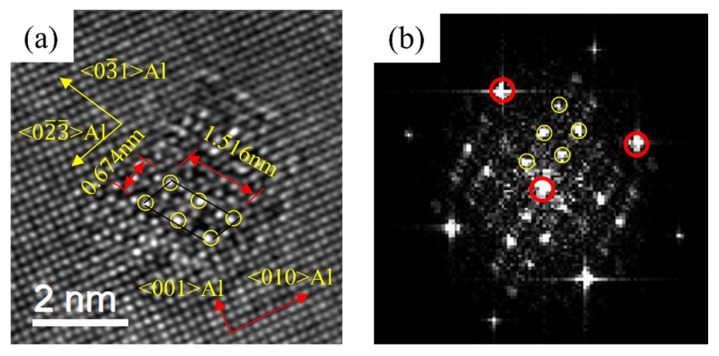
HRTEM (**a**) and FFT (**b**) patterns of the β″ phase of 0.18Cu alloy after peak aging at 175 °C for 10 h.

**Figure 11:**
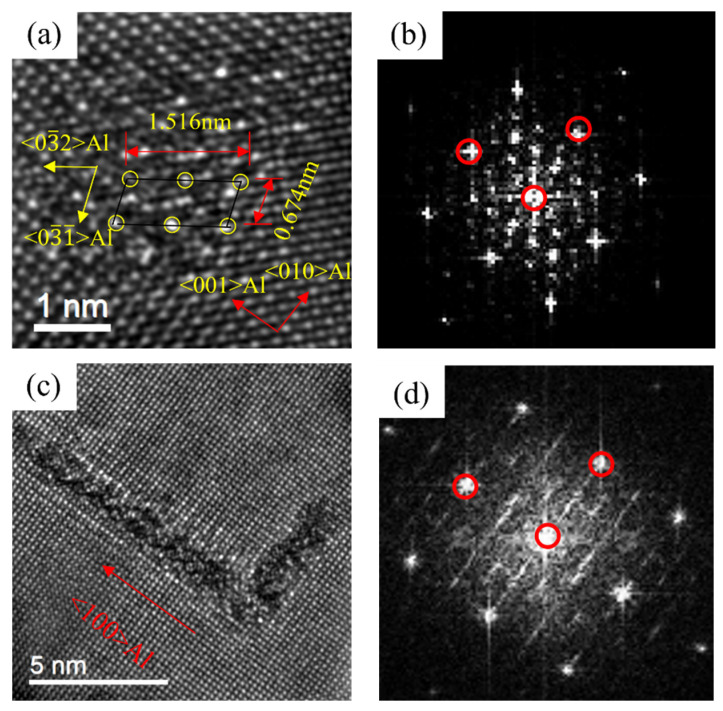
HRTEM and FFT patterns of the precipitates of 0.37Cu alloy after peak aging at 175 °C for 8 h. (**a**,**b**) β″; (**c**,**d**) L.

Figure 9, Figure 10 and Figure 11 are the HRTEM and the FFT patterns of the three alloys after peak aging. It can be found that that β″ phase was precipitated in the peak aging stage of the three alloys. The crystal structure of the precipitates was mainly a bottom-centered monoclinic structure. The crystal parameters were: a = 1.516 nm, c = 0.674 nm, γ = 105.26°. It was completely coherent with the substrate along the b-axis, and the other two axes were parallel to the directions of 〈310〉_Al_ and 〈230〉_Al_. The relationship with the Al matrix was mainly semi-lattice. This was consistent with the β“ phase parameter in the literature [22]. Figure 9 shows the precipitates of 0Cu alloy at the peak aging stage. It can be seen that the precipitates mainly grew along the 〈100〉_Al_. Figure 10 exhibited the precipitates of 0.18Cu alloy at the peak aging stage. Only the β″ phase was found in the alloy. Figure 11 displayed the precipitates of 0.37Cu alloy at the peak aging stage. Through calibration and comparison, a lath-like precipitate was found in the 0.37Cu alloy, and its long edge was elongated with 〈100〉_Al_. In FFT patterns, the diffraction streaks of precipitates were parallel to the direction of 〈100〉_Al_, which was the same as the studies of Torsæter M [30] and Jia Z H [32]. Therefore, it was determined to be the L phase. As a precursor to the Q′ phase, the L phase had good thermal stability [39]. In addition to being distinguished from the lattice parameters, the two precipitates can also be intuitively identified from the morphology, in which the L phase was elongated along the direction of 〈100〉_Al_, while the Q′ phase was elongated in the direction of 〈510〉_Al_.

Table 6 shows the relevant statistical information of the β″ phase in the alloys under the peak aging state. It was found that the size of the β″ phase decreased with the Cu content increasing, but the number density and volume fraction raised. From the quantitative analysis result, it can be seen that Cu promoted the precipitation of the β″ phase at the peak aging stage. As shown in Figure 2 and Figure 3b, the mechanical property of the alloys was significantly improved with the increase in Cu at the peak aging stage. Additionally. the strength of the 0.37Cu alloy was the highest. That indicated that the addition of Cu enhanced the mechanical properties of Al-1.2Mg-1.2Si-(xCu) alloy by promoting the precipitation of the strengthened phase. In comparison with the initial aging stage, the number of precipitates had increased. So as that, the mechanical properties of the alloys at the peak aging stage were higher than that at the initial aging stage.

#### 3.2.3. Precipitates of Alloys at the Over-Aged Stage

**Figure 12:**
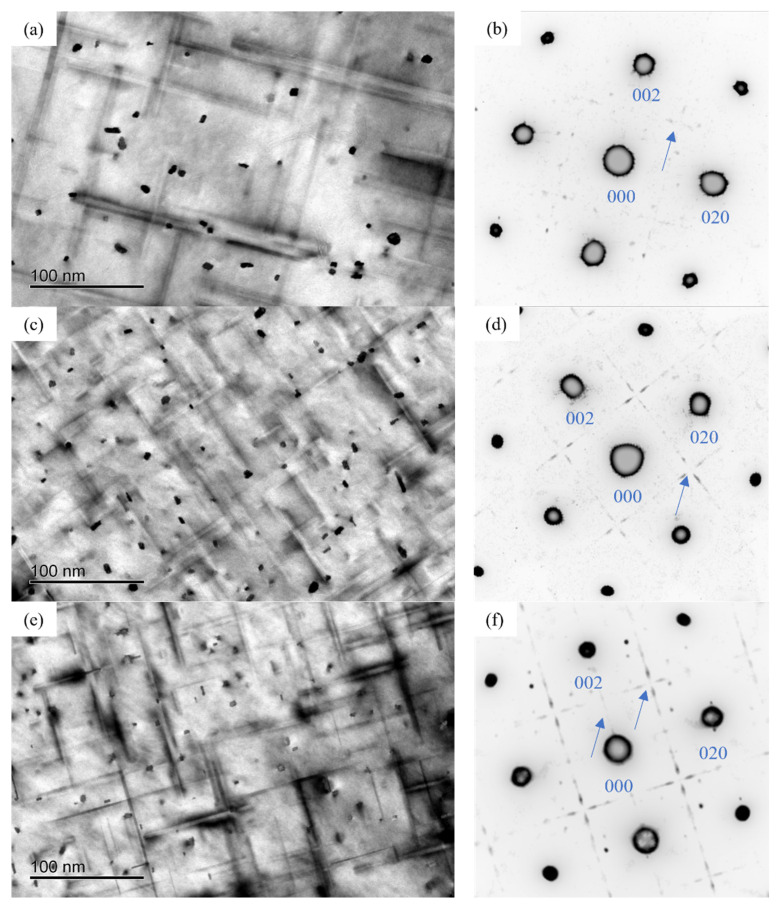
BF image and SAED patterns after over-aging at 175 °C for 72 h of three alloys. (**a**,**b**) 0Cu alloy; (**c**,**d**) 0.18Cu alloy; (**e**,**f**) 0.37Cu alloy.

Figure 12 shows the BF images and SAED patterns of the alloys after over-aging at 175 °C for 72 h. It can be found that the needle-like and lath-like precipitates grew in the direction of 〈100〉_Al_. In addition to the diffraction spots of Al matrix, the diffraction streaks aligned along the 〈100〉_Al_ direction were obviously observed. In the over-aging stage, the size of the precipitated phase decreased with the increase in Cu content. Compared with the peak aging stage, the precipitates coarsened obviously after over-aging at 175 °C for 72 h.

**Figure 13:**
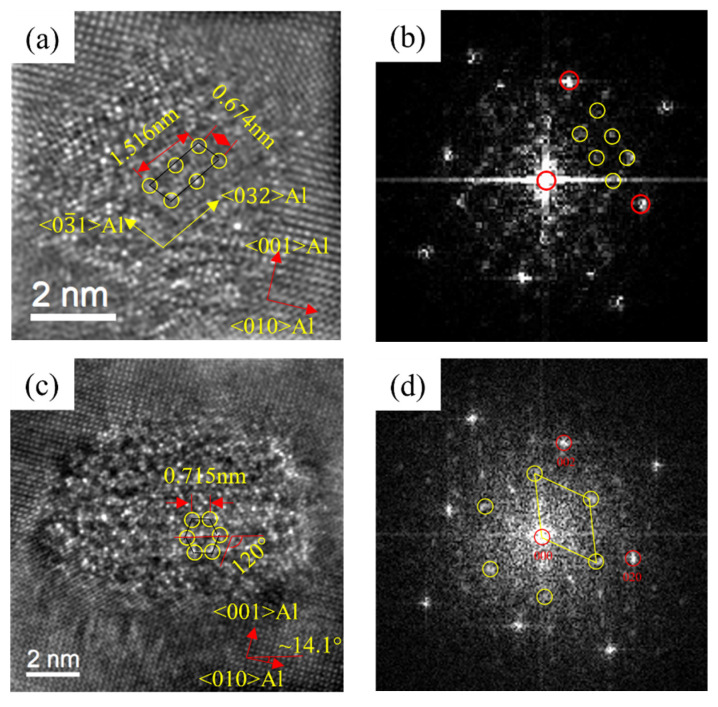
HRTEM and FFT patterns of 0Cu alloy precipitated after over-aging at 175 °C for 72 h. (**a**,**b**) β″; (**c**,**d**) β′.

Figure 13 revealed the HRTEM and FFT of the precipitates of 0Cu alloy after over-aging at 175 °C for 72 h. By calibrating the lattice parameters of the precipitates, two types of precipitates of 0Cu alloys can be found. In Figure 13, a noodle-like phase with the crystal parameters of a = 1.516 nm, c = 0.674 nm and γ = 105.26° was found. Its axis was parallel to 〈320〉_Al_ and 〈310〉_Al_, which was the same to the found of Andersen S J [22] and determined to be β″ phase. Moreover, a rod-like phase with the crystal parameters of a = b = 0.715 nm, γ = 120° was found. The ORs between this precipitate and Al matrix was 14.1°. This was consistent with the research of Vissers R [40]. It can be determined that the rod-like phase was β′ phase. Consequently, the noodle-like β″ phase and rod-like β′ phase were formed at the over-aged stage of the 0Cu alloy.

**Figure 14:**
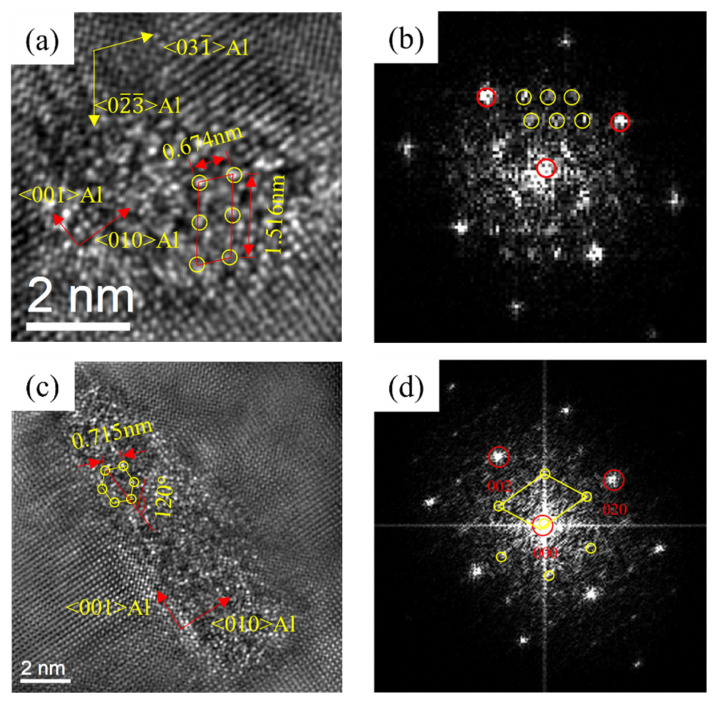
HRTEM and FFT patterns of 0.18Cu alloy after over-aging at 175 °C for 72 h. (**a**,**b**) β″; (**c**,**d**) β′.

Figure 14 displays the HRTEM and FFT patterns of the precipitates of 0.18Cu alloy after over-aging at 175 °C for 72 h. There were two types of precipitates in 0.18Cu alloy. One had the same crystal parameters and ORs with the β″ phase. Another was a lath-like phase, and it had a crystal parameter of a = b = 0.715 nm, γ = 120°. The ORs between the precipitate and Al matrix was 0°. The studies of Vissers R [40] indicated that the lath-like β′ phase had a constant β′-angle of 0°. It was consistent with the result of this study. Therefore, the β″ phase and lath-like β′ phase were precipitated at the over-aged stage of the 0.18Cu alloy.

**Figure 15:**
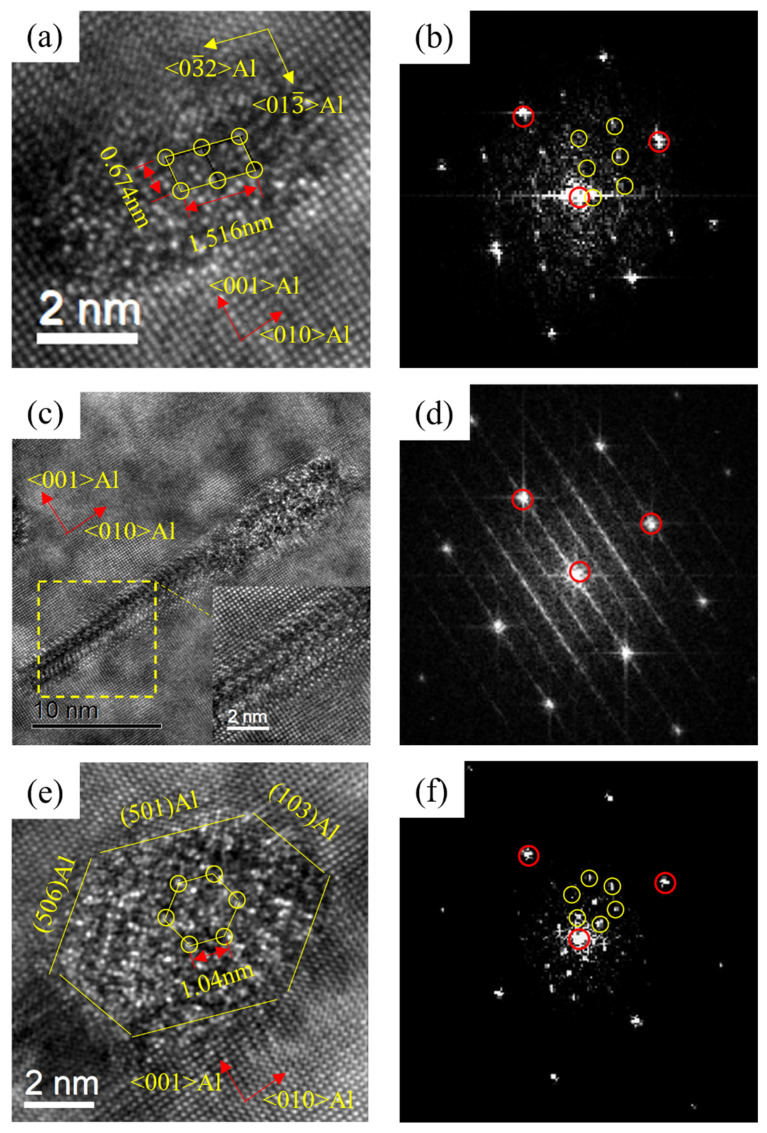
HRTEM and FFT patterns of 0.37Cu alloy after over-aging at 175 °C for 72 h. (**a**,**b**) β″; (**c**,**d**) L; (**e**,**f**) Q′.

Figure 15 shows the HRTEM and FFT patterns of the precipitates of 0.37Cu alloy after over-aging at 175 °C for 72 h. There were three types of precipitates in 0.37Cu alloy. The first precipitate was β″ phase with a crystal parameter of a = 1.516 nm, c = 0.674 nm, and γ = 105.26°, and its axis was parallel to 〈320〉_Al_ and 〈310〉_Al_. The second phase had a lath-like microstructure, and it grew parallel with 〈100〉_Al_. It was consistent with the study about the L phase of Torsæter M [30] and Jia Z H [32]. In addition, a precipitate with a hexagonal shape was found. It had a crystal parameter of a = b = 1.04 nm, γ = 120°, and its axis was parallel with (506)_Al_, (501)_Al_, and (103)_Al_ separately. It was consistent with the study about the Q′ phase of Weng Y Y [26]. As a result, the precipitates of β″ phase, L phase, and Q′ phase were formed at the over-aged stage. As shown in Figure 2 and Figure 3c, the mechanical properties of the alloys were significantly improved with the increase in Cu content at the over-aged stage. The Q′ phase was a metastable version of the quaternary equilibrium phase Q phase, which played an important role in the precipitation hardening of Al-Mg-Si-Cu alloy [24,41]. In consequence, the strength of 0.37Cu alloy was the highest at the over-aged stage. Therefore, β″ phase, lath-like L phase, and Q′ phase were precipitated at the over-aged stage of the 0.18Cu alloy.

In comparison with the initial aging stage and peak aging stage, the coarsening of precipitates can be directly observed from the bright field image. Additionally, the mechanical property decreased. Consequently, in the over-aging stage, the coarsening would have a bad effect on the mechanical property of the alloy.

## 4. Discussion

Aging hardening was the main strengthening mechanism of Al-Mg-Si-(xCu) alloys [31], and the effective ways of aging hardening depended largely on the type, number, size, and distribution of nanoscale precipitates [42]. The precipitates of the three alloys after early aging were characterized by TEM and HRTEM. The results showed that GP zones and pre-β″ were mainly precipitated in the early aging stage. Murayama [35] found that the precipitation of GP zones was quite important to the property of the alloy. The formation of the GP zones could produce a certain refinement effect. Therefore, it was necessary to compute the size, number density, and volume fraction of precipitates in the early stage. Because the content of pre-β″ was too small, it was difficult to observe from the bright field image. The precipitate in the statistical process was regarded as GP zones. Results showed that the diameter of the GP zones decreased slightly, and its number density and volume fraction increased, which indicated that the addition of Cu promoted the precipitation of GP zones. Meanwhile, the Vickers hardness and tensile strength of the alloy were significantly increased with the increase in Cu element in the initial aging stage. In the early stage of aging, due to the strong interaction between Cu and Mg, the formation of Al-Mg-Si-Cu clusters can be the nucleation site to GP zones. This also accelerated the formation of the finely distributed GP zones and ultimately increased the Vickers hardness and tensile strength of the alloy after the early aging process [43,44]. As a consequence, the main reinforcement mechanism of Cu addition was precipitation strength. 

With the extension of the aging time, the alloys reached the peak aging point successively. With the increase in Cu, the time to reach peak aging was shortened and the corresponding peak Vickers hardness was increased. The corresponding tensile strength which both exceeded 420 MPa of peak aging stage increased significantly with the increase in Cu. Through the characterization of TEM at the peak aging stage of the alloy, it can be seen that the addition of Cu can significantly affect the aging precipitation behavior of the alloy. Firstly, the addition of the Cu element changed the precipitation sequence of the alloy, and the main precipitates at the peak aging stage of 0Cu and 0.18Cu alloy were the β″ phase, while the main precipitates after peak aging of 0.37Cu alloy were β″ phase and L phase. Through the quantitative characterization of the precipitates, it can be found that with the increase in Cu content, the size of the precipitates was decreased, and the number density and volume fraction were increased. It was shown that the addition of Cu promoted the precipitation of the strengthening phase in the alloy and made the precipitates smaller. The finely dispersed precipitates can significantly prevent the dislocation from moving, thus enhancing the strength of the alloy. Therefore, the Vickers hardness of the alloy which added Cu was the highest, so as to tensile strength. Compared with the initial stage of aging, the quantity density and volume fraction of precipitates in peak aging were obviously increased. In addition, the β″ phase was the main strengthening phase during peak aging, while the main strengthening phase was GP zones in the initial aging stage. The strengthening effect of β″ was higher than that of GP zones. Synthesizing the quantity and strengthening effect, the strength of the alloys at peak aging was significantly higher than that at the initial aging stage.

The characteristic results manifested that the main precipitates in 0Cu alloy and 0.18Cu was β″ phase and β′ phase. The main precipitates in 0.37Cu alloy were β″ phase, L phase, and Q′ phase which had good thermodynamic stability. It was found that three alloys retained part of the peak aging precipitates after over-aging, and the over-aging phase was precipitated at the same time. The over-aging phases precipitated in the 0Cu alloy and 0.18Cu alloy were β′ phase, while the over-aged phase of the 0.37Cu alloy was Q′ phase. It can be speculated that when the addition of Cu was 0.37, the precipitation of the Q′ phase was promoted. Because the precipitates in the over-aging stage were too coarse, the statistical results would produce a large error. Consequently, the precipitates can only be qualitatively analyzed through the light field images directly. The mechanical property test result showed that the strength and Vickers hardness of the alloy still enhanced with the increase in Cu content at the over-aging stage. In addition, compared with the peak aging precipitates, over-aging precipitates coarsened obviously. The fine precipitate was conducive to nailing the dislocation and improving the properties of the alloy. Therefore, the performance of over-aging was significantly decreased compared with peak aging.

## 5. Conclusions

In this investigation, Cu addition on precipitation hardening behavior and mechanical property of Al-1.2Mg-1.2Si-(xCu) alloy was studied, and the following conclusions were reached:

1. Cu enhanced the artificial aging hardening response and improved the tensile properties of the Al-1.2Mg-1.2Si-(xCu) alloy. With the increase in Cu, the time to reach peak aging decreased which was 12 h of 0Cu alloy, 10 h of 0.18Cu alloy, and 8 h of 0.37Cu alloy. In addition, their tensile strength was 421 MPa, 448 MPa, and 459 MPa, respectively.
2. The addition of 0.37Cu changed the type of precipitates at the peak and over-aging stage and the aging precipitation sequence of Al-1.2Mg-1.2Si-(xCu) alloy. The precipitation sequence of 0Cu and 0.18Cu alloy was SSSS→GP zones/pre-β″→β″→β″ + β′, 0.37Cu alloy was SSSS→GP zones/pre-β″→β″ + L→β″ + L + Q′.
3. Cu addition promoted the precipitation of enhanced phases. With the increase in Cu, the number density of precipitates was increased from 0.23 × 10^23^/m^3^ to 0.73 × 10^23^/m^3^, and the volume fraction of precipitates was increased from 0.27% to 0.59% in the initial aging stage. The number density of precipitates was increased from 1.9 × 10^23^/m^3^ to 5.5 × 10^23^/m^3^ and the volume fraction of precipitates was increased from 4.05% to 5.36%. Accordingly, the tensile strength of the alloy was promoted with the increase in Cu.

## Figures and Tables

**Table 1:** The crystal parameters and ORs of precipitates reported in Al-Mg-Si(-Cu) alloys.

Precipitate	Crystal Parameters (nm)	Orientation Relationship	Refs.
β″	a = 1.516 nm, b = 0.405 nm, c = 0.674 nm, γ = 105.26°	(010)_β″_//{100}_Al_; [001]_β″_//<310>_Al_, [100]_β″_//<230>_Al_	[18,23]
Q′	a = b = 1.04 nm, c = 0.405 nm, γ = 120°	[0001]_Q′_//[001]_Al_, [1$\bar{2}$10]_Q′_//[130]_Al_	[24,28]
L	a = 1.032 nm, b = 0.405 nm, β = 100.9°, c = 0.81 nm	/	[28,30,32]
C	a = 1.032 nm, b = 0.81 nm, c = 0.405 nm, γ = 101°	(001)_C_//(001)_Al_, [100]_C_//[100]_Al_	[20,21,22,23,24,25,26,27,28,29,30]
β′	a = b = 0.715 nm, c = 1.215 nm, γ = 120°	[001]_β′_//[001]_Al_, [2$\bar{1}\bar{1}$0]_β′_//[310]_Al_	[26]
θ′	a = 0.404 nm, c = 0.58 nm	(200)_θ′_//(200)_Al_, [010]_θ′_//[010]_Al_	[31]

**Table 2:** Chemical composition table of the Al-Mg-Si-(xCu) alloys.

Alloy	Mg	Si	Cu	Mn	Cr	Zn	Fe	Ti	Al
0Cu	1.20	1.23	/	0.42	0.16	0.12	0.028	0.020	Bal.
0.18Cu	1.16	1.20	0.18	0.43	0.17	0.12	0.016	0.020	Bal.
0.37Cu	1.13	1.20	0.37	0.42	0.17	0.12	0.026	0.020	Bal.

**Table 3:** Vickers hardness of alloys at different aging stages.

Alloy	Vickers Hardness (HV).			
	Solution Treated	Initial Aging	Peak Aging	Over-Aging
0Cu	70	95	134	100
0.18Cu	73	104	140	115
0.37Cu	74	123	142	119

**Table 4:** Mechanical properties of extruded aluminum alloy.

Alloy	Tensile Strength, MPa	Range, MPa	Yield Strength, MPa	Range, MPa	Elongation, %	Range, %
6013-T6511 [34]	393	/	365	/	11	/
6056-T6511 [34]	393	/	372	/	10	/
0Cu-T6	421	3	390	4	12.2	1.2
0.18Cu-T6	448	2	427	5	12.8	2.8
0.37Cu-T6	459	5	426	7	13.2	2.0

**Table 5:** Statistical information of GP zones of the alloys after aging at 175 °C for 0.5 h.

Alloy	Diameter (nm)	Number Density (10^23^/m^3^)	Volume Fraction (%)
0Cu	2.82	0.23	0.27
0.18Cu	2.53	0.33	0.28
0.37Cu	2.49	0.73	0.59

**Table 6:** Statistical information of the β″ phase of the alloy at peak aging stage.

Alloy	Cross Area (nm^2^)	Length (nm)	Number Density (10^23^/m^3^)	Volume Fraction (%)
0Cu	6.61	32.23	1.9	4.05
0.18Cu	5.69	26.11	3.1	4.61
0.37Cu	4.79	10.33	5.5	5.36
